# Experiences of Loneliness Associated with Being an Informal Caregiver: A Qualitative Investigation

**DOI:** 10.3389/fpsyg.2017.00585

**Published:** 2017-04-19

**Authors:** Konstantina Vasileiou, Julie Barnett, Manuela Barreto, John Vines, Mark Atkinson, Shaun Lawson, Michael Wilson

**Affiliations:** ^1^Department of Psychology, University of BathBath, UK; ^2^Department of Psychology, University of ExeterExeter, UK; ^3^Lisbon University Institute (ISCTE-IUL/CIS)Lisbon, Portugal; ^4^School of Design, Northumbria UniversityNewcastle, UK; ^5^Department of Computer and Information Sciences, Northumbria UniversityNewcastle, UK; ^6^School of the Arts, English and Drama, Loughborough UniversityLoughborough, UK

**Keywords:** loneliness, social isolation, informal caregivers, qualitative interviews, experiences, United Kingdom

## Abstract

Although providing care to a family member or friend may provide psychological benefits, informal (i.e., unpaid) caregivers also encounter difficulties which may negatively affect their quality of life as well as their mental and physical health. Loneliness is one important challenge that caregivers face, with this psychological state being associated with morbidity and premature mortality. Although previous research has identified loneliness as an issue associated with being an informal caregiver, there is a paucity of evidence that attempts to understand this phenomenon in depth. This study aimed to examine informal caregivers' reflections on, and accounts of, experiences of loneliness linked to their caregiving situation. As part of a cross-sectional, qualitative study, sixteen semi-structured interviews were conducted with 8 spousal caregivers, 4 daughters caring for a parent, 3 mothers caring for a child (or children), and 1 woman looking after her partner. The cared-for persons were suffering from a range of mental and physical health conditions (e.g., dementia, frailty due to old age, multiple sclerosis, depression, autism). Data were analyzed using an inductive thematic analysis. Experiences of loneliness were described by reference to a context of shrunken personal space and diminished social interaction caused by the restrictions imposed by the caregiving role. Loneliness was also articulated against a background of relational deprivations and losses as well as sentiments of powerlessness, helplessness, and a sense of sole responsibility. Social encounters were also seen to generate loneliness when they were characterized by some form of distancing. Though not all sources or circumstances of loneliness in caregivers are amenable to change, more opportunities for respite care services, as well as a heightened sensibility and social appreciation of caregivers' valued contributions could help caregivers manage some forms of loneliness.

## Introduction

Loneliness and social isolation are increasingly recognized as important societal challenges. Approximately 15% of adults in the UK aged 16–79 years old report high levels of loneliness in their daily life with double this percentage in people aged over 80 (Thomas, 2015). Loneliness is defined as “the unpleasant experience that occurs when a person's network of social relations is deficient in some important way, either quantitatively or qualitatively” (Perlman and Peplau, 1981, p. 31). Despite being associated with social isolation—a situation which refers to a quantitatively diminished social network—loneliness is considered to be a distinct concept which necessitates a *subjective* and *negative* evaluation of the existing status of one's social network (Yang and Victor, 2011). Depending on the nature of the social deficit that is involved, two types of loneliness have been proposed (Weiss, 1973): *Emotional loneliness* occurs when one lacks intimate and close relationships and *social loneliness* results from inadequate integration with social networks (e.g., derived from employment, kin, friendships, or neighborhood), or rejection by the broader community (e.g., residing in an unaccepting community). Loneliness can negatively influence higher-order cognitive processes (e.g., attention, memory, emotional regulation, logical reasoning; Cacioppo and Hawkley, 2009) and manifest affectively in desperation, depression, boredom and self-deprecation (Rubenstein and Shaver, 1982). Behaviorally, lonely people tend to encounter others in a more self-absorbed and less socially effective manner than non-lonely individuals (Heinrich and Gullone, 2006).

Given the crucial role of social relationships and social support networks in health and well-being (Cohen, 2004; Holt-Lunstad et al., 2010), a substantial body of research has examined the links between loneliness and social isolation and physical and mental health outcomes. Research suggests that loneliness and social isolation are associated with increased likelihood of mortality (Pantell et al., 2013; Holt-Lunstad et al., 2015; Holwerda et al., 2016), increased risk of developing coronary heart disease, stroke (Valtorta et al., 2016) high blood pressure (Hawkley et al., 2010) and engagement with unhealthy behaviors (e.g., smoking, alcohol consumption) (Lauder et al., 2006; Nieminen et al., 2013). Loneliness is also related to poor mental health outcomes (for a review see Heinrich and Gullone, 2006), such as depression (Cacioppo et al., 2010; Teo et al., 2013), deliberate self-harm (Rönkä et al., 2013), increased risk of dementia (Holwerda et al., 2014), and Alzheimer's disease (Wilson et al., 2007). It is also associated with increased frequency of older adults' visits to their doctor (Ellaway et al., 1999; Gerst-Emerson and Jayawardhana, 2015), thus impacting on healthcare costs.

Despite agreement about the subjective nature of the phenomenon of loneliness, most research in the area has historically tended to be quantitative, limiting understanding of the lived experience of loneliness. In response to this, more recent research endeavors employing qualitative methods have been directed to an examination of the ways people experience and make sense of loneliness (Dahlberg, 2007). Given the emphasis on loneliness in *older age*, most qualitative (or mixed-method) research has been conducted with older people (e.g., Graneheim and Lundman, 2010; Stanley et al., 2010; Hauge and Kirkevold, 2012; Tiilikainen and Seppänen, 2016). However, loneliness has been explored in other populations, such as people with mental health problems (e.g., Lindgren et al., 2014), those with intellectual disabilities (e.g., McVilly et al., 2006), school-aged children (e.g., Berguno et al., 2004), adolescents (e.g., Ruiz-Casares, 2012), and students (e.g., Sawir et al., 2008). This literature reveals the complex, diverse, and multifaceted nature of the experience of loneliness that is contingent on contextual and person-related factors.

Important life transitions that induce changes in one's existing or desired social relations and interactions can precipitate the onset of loneliness (Perlman and Peplau, 1981; Peplau and Perlman, 1982). Taking on a caregiving role often constitutes such a transition; a major life event that is likely to disrupt one's status of social relations. An informal caregiver is defined as the person who (in contrast to professional caregivers) provides unpaid care to a family member, partner, friend or neighbor because of long-term physical or mental ill health, disability, or problems related to old age. The caregiver can either co-habit with the cared-for person or not and care provision can range from a few hours per week to round-the-clock (Carers UK, 2015b). Informal caregivers constitute a sizeable minority of the general population. According to 2011 census data, 5.8 million people in England and Wales provided informal care to a family member, friend, or neighbor, representing just over 1 in 10 of the population (Office for National Statistics, 2013). Indeed everyone is likely to become a caregiver at some point in their lives as care demand is estimated to grow in the future (Office for National Statistics, 2013). For example, estimates suggest that 9 million caregivers will be needed in the UK by 2037 (Carers UK, 2015b). The majority of caregivers in Britain are people of working age with the peak age of caregiving being between 50 and 64 years old. 58% of caregivers are female, 42% are male and the majority look after their parents or parents-in-law (40%) or their spouse or partner (26%) (Carers UK, 2015b).

Despite the identification of some psychological benefits that arise from providing care to a family member or friend, such as a sense of greater closeness toward the cared-for individual or enhanced sense of purpose and meaning in life (Kramer, 1997; Cohen et al., 2002; Mackenzie and Greenwood, 2012), the strain of caregiving role places this population at risk of poor psychological and physical health (Pinquart and Sörensen, 2003; Vitaliano et al., 2003). Research indicates that caregivers have higher levels of stress and depression and lower levels of subjective well-being than non-caregivers (Pinquart and Sörensen, 2003; Verbakel, 2014) and encounter a greater risk of developing physical health problems (Vitaliano et al., 2003), particularly those caregivers who are psychologically distressed and/or face behavioral difficulties of the cared-for person (Pinquart and Sörensen, 2007). The caregiving role can also restrict caregivers' participation in social activities (Clark and Bond, 2000) thus limiting the psychological benefits that accessing social support offers (Cannuscio et al., 2004) as well as the opportunity for a satisfying social life.

Though prevalence studies are lacking, it has been estimated that 8 in 10 caregivers in the UK have felt lonely or socially isolated as a result of their caregiving situation (Carers UK, 2015a). Supporting these estimates, qualitative research with men caring for a spouse or a parent has identified feelings of loneliness to be one of the significant elements of the caregiving experience (Parsons, 1997; Siriopoulos et al., 1999) and cross-sectional research shows that caregivers report higher levels of loneliness than non-caregivers (Beeson, 2003). Loneliness in the caregiving population appears to be qualitatively different from the one reported by the general population with caregivers scoring higher levels on the aspect of self-alienation (Rokach et al., 2007). Characteristics such as lower educational level, low self-efficacy, poorer physical health and being a female are predictive of loneliness in caregivers (McRae et al., 2009; Soylu et al., 2016). Finally, loneliness in caregivers is associated with psychological distress (Chukwuorji et al., 2016) and significantly predicts depression (Beeson et al., 2000; Beeson, 2003) and low quality of life (Ekwall et al., 2005).

The present study used a qualitative methodology to examine how informal caregivers, encountering a diversity of caregiving situations, experience and make sense of loneliness linked to their caregiving situation. Given the significant links, as noted above, between loneliness, health and well-being as well as the health-related challenges that caregivers face more broadly, by virtue of the stressors of their caregiving context, it is timely to pay closer attention to caregivers' experiences of loneliness.

## Methods

### Study context and design

The study reported here is part of a larger mixed-method research project that examines experiences of loneliness—defined as the distressing experience deriving from a discrepancy between one's desired and actual levels of social relations (Perlman and Peplau, 1981)—in people whose social relations are likely to alter and be disrupted on account of work situations (i.e., lone and remote working) and major life changes and transitions (i.e., assuming a caregiving role; moving away from home to study). This research project also seeks to investigate how digital technologies can facilitate social exchanges that are characterized by empathy and trust that might, in turn, alleviate experiences of loneliness and foster meaningful and satisfying social connections. The findings presented in this paper come from the cross-sectional qualitative exploratory phase of the project during which 45 semi-structured interviews were conducted with informal caregivers (*n* = 16), students (*n* = 15), and remote and lone workers (*n* = 14). This study was carried out in accordance with the recommendations of the British Psychological Society. The study protocol received ethical approval from the Department of Psychology (Ethical approval reference number: 15–149) at the University of Bath and the Ministry of Defence Research Ethics Committee (Application number: 620/MoDREC/14). All participants gave written informed consent in accordance with the Declaration of Helsinki.

### Study population, sampling, and recruitment

The study population of the research reported in this article consisted of informal caregivers who self-identified as experiencing some form of loneliness and/or social isolation associated with their responsibilities of providing care and who reported that they had significantly limited their activities as a result of their caregiving role. A non-probability purposive sampling approach was thus adopted that allowed us to recruit caregivers who were likely to provide rich and in-depth accounts of experiences of loneliness. Participants for this study were recruited with the assistance of a voluntary organization in the South West England. Around 3,500 caregivers are registered with this organization, of which 500 are young caregivers. Of the adult caregivers, 69% are female and 31% are male. The average age of the adult caregivers registered with the organization is 57 years old.

Two-hundred research invitation letters were sent to caregivers registered with the voluntary organization describing briefly the study. Seventy-five caregivers expressed an interest in finding out more about the research by returning their contact details to the researchers, using a pre-paid envelope. Prospective participants were then provided with: (a) a *Participant Information Sheet* explaining in greater detail the aims of the study, the research process, and their rights as research participants; (b) an *Informed Consent Form* to be signed prior to the interview; and (c) a *short Screening Questionnaire* (please refer to Supplementary Material: Screening questionnaire). The screening questionnaire was used to collect basic demographic data (i.e., gender, age, educational level, nationality, and marital status), information about the caregiving situation (i.e., caregiver's relationship to the cared-for person; duration of caregiving situation; number of hours providing care on a “typical” day; access to respite care; and extent to which the caregiver had limited or stopped activities as a result of the caregiving role) and information about the use of communication technologies, including any potential use of digital technologies. Twenty-eight caregivers returned their screening questionnaire. From those, 16 caregivers who replied back within the timeframe of the data collection period and reported in their screening questionnaire significant activity restriction due to the caregiving situation (“somewhat” or “very much”) were invited to the interview to meet sampling requirements.

### Participants

In total 16 caregivers (11 women; *Mean* age = 63 years old, *min* = 24, *max* = 91; 12 participants ≥ 59 years old) were interviewed. Eight participants were spousal caregivers; one woman was caring for her partner; four caregivers were looking after a parent; and three caregivers were mothers caring for their child or children with significant health problems. Seven participants were caring for somebody with dementia, six people were looking after someone with primarily a physical illness (one case, physical illness, and depression), and the three mothers looked after children with a psychological or a developmental disorder. All caregivers were living with the cared-for person, except for one mother who lived separately from her adult daughter at the time of the interview. Four of the participants were assisted by professional caregivers at home and three regularly accessed respite services. Fourteen caregivers were British (two did not report their nationality); six caregivers had received higher education; seven had received education to less than university degree level; and three participants reported no qualifications. Table 1 presents the participants' demographic characteristics and the health status of the cared-for person. In the analysis section below, the interview identification code, the participant's gender, age and relationship to the cared-for person are provided after each quotation to contextualize the accounts.

**Table 1:** **Caregivers' gender and age by category on the basis of the relationship to the cared-for person and health status of care recipients**.

**Gender**	**Age**	**Health status of cared-for person**
**CARING FOR A SPOUSE:** *n* = 8		
Female (wives) = 3;	*Mean* = 73	• Dementia (6 care recipients)
Male (husbands) = 5	*Min* = 41	• Multiple sclerosis (1 care recipient)
	*Max* = 91	• Osteoarthritis, rheumatoid arthritis, and fibromyalgia (1 care recipient)
**CARING FOR A PARENT:** *n* = 4		
Female (daughters) = 4	*Mean* = 65	• Dementia (1 care recipient)
	*Min* = 60	• Physical illnesses and frailty due to old age (3 care recipients)
	*Max* = 69	
**CARING FOR A CHILD:** *n* = 3		
Female (mothers) = 3	*Mean* = 51	• Bipolar disorder (1 adult child);
	*Min* = 46	• Attention deficit hyperactivity disorder and high functioning autism;
	*Max* = 59	• Developmental disorder (1st child) and autism (2nd child)
**CARING FOR A PARTNER:** *n* = 1		
Female (partner)	24 years old	• Depression and physical illnesses related to infection and the operation of the immune system

### Data collection

Date were collected in October and November 2015. In accordance with caregivers' wishes, 11 interviews were conducted at participants' home, 3 at the University of Bath and 1 at the premises of the voluntary organization through which participants were recruited. A semi-structured interview protocol was developed to guide the conversations (please refer to Supplementary Material: Interview objectives and protocol). The interview was divided into two main parts. In the first section, participants were invited to discuss their caregiving situation (e.g., how they took up their caring responsibilities and what these included; duration of the caring situation; the main challenges they have faced; the impact of the caring situation on caregivers' life; available support from family, friends and outside organizations and agencies). The second part of the interview explored experiences of loneliness and social isolation as well as any management strategies the caregivers had developed to cope with these experiences. To close the interview, participants were invited to add any final thoughts or observations they wished to make around experiences of loneliness in caregivers more broadly. The interviews lasted on average 1 h (shortest = 25 min; longest = 90 min), were audio-recorded and were then transcribed verbatim. At the end, participants were provided with a debrief sheet which included a list of support contacts. Participants were also offered a High Street voucher as a token of appreciation for contributing to the research. The first author, a psychologist by education with extensive experience in qualitative research, conducted the interviews.

### Analytic approach

A thematic analysis was adopted to analyze the data using the six-phase process suggested by Braun and Clarke (2006, 2012). Thematic analysis is a suitable analytic approach for identifying “patterns of meanings across a data set” in a systematic manner (Braun and Clarke, 2012, p. 57). Moreover, this analysis was informed by a critical realist epistemological standpoint (Bhaskar, 1989). Situated between a naïve realist and a purely relativist position, critical realism assumes that language is constitutive of social realities and meaning. Nevertheless, extra-discursive elements—in particular material conditions—also impact upon meaning and subjectivity by delimiting which discursive constructions are more or less dominant, and thus more or less available (Sims-Schouten et al., 2007). Taking a critical realist standpoint allowed us to examine caregivers' discursive constructions of experiences of loneliness while also being attentive to the significance and influence of the material contexts within which they operated (e.g., the cared-for person's health status). Though pure induction is unattainable, as the researcher can never completely escape their own pre-conceptions, this analysis largely employed a bottom-up, data-driven approach, which sought to empirically ground how participants themselves made sense of their experiences of loneliness.

## Results

Four main themes were identified from the analysis: (a) Loneliness was located within a context of *shrunken personal space* and *diminished social interaction* resulting from the restrictions posed by the caregiving role; loneliness was articulated against (b) a background of *relational deprivations* and *losses*, as well as (c) social encounters characterized by some form of *distancing* and *separateness*; (d) finally, sentiments of *powerlessness, helplessness* and a sense of *sole responsibility* were considered to induce feelings of loneliness.

### Theme 1: shrunken personal space and diminished social interaction

Experiences of loneliness in informal caregivers were often seen to be linked to the restrictions that the caregiving situation imposed. Participants commonly articulated how their everyday life was characterized by limited freedom to define the management of their time and choice of space, by a lack of spontaneity, and with little opportunity to be free from concern. The needs and well-being of the cared-for person were a constant pre-occupation and priority, whilst time away from the care receiver required considerable planning on the part of the caregiver.

*I can't do so much as I used to do. I can't leave him in the house, I can't go off and leave him, he's always got to be with me. My life has narrowed down a bit* (P05: Female, 81, cares for husband).

The sense of restriction was very intense in some instances, as illustrated through the use of imprisonment as an analogy.

*That was lovely to get out and just be out, you know? We were doing something for ourselves, you know? It was like we'd been let out a cage or something?!* (P07: Female, 60, cares for mother).

Some participants described that they missed the freedom and spontaneity to be able to meet friends outside the home for as long as they wanted to and whenever this opportunity arose. And although participants expressed the view that this restriction could to some extent be counterbalanced by inviting friends to their house, they simultaneously acknowledged that this sort of social interaction has limitations as it depends on other people's availability and willingness to visit them as well as on the cared-for person's sense of comfort and receptiveness to regularly have visitors at home.

*I miss going out with my friends, they all go all over the place still and they're all widows mainly. They do what they want and go out and enjoy themselves, which is right, but I can't do that because I can't leave him and I haven't got anyone to come and look after him. So that's difficult, I find that really difficult that I can't go out just when I want to* (P01: Female, 82, cares for husband).

In discussing their views as to whether loneliness might be a common challenge among the caregiving population, the participants considered that feelings of loneliness are prevalent in caregivers due to the disconnection and social isolation that the caregiving situation induces. The simile of new mothers who are entirely committed and devoted to the needs of the new-born baby was used by one participant to explain why caregivers might be particularly susceptible to loneliness.

*Because you [‘re] cut off, your life is so involved with that person, you're cut off from so much because you just are so involved. It's very much like a mum who's caring on her own or even if her partner's away at work and she's got a new baby, she's so involved in what she's got to do, she may not have the friends or the family or the opportunity to link in so in the same way, it's like that* (P08: Female, 69, cares for father).

Loneliness was exacerbated by the requirement of constant attentiveness to the cared-for person, which significantly shrank the caregiver's real and psychological space. The boundaries between the self and the other were forced to be drawn in ways that limited the fulfillment of the caregiver's social needs and desires. This created tension between the requirements of the caregiving role and the caregiver as a person.

*You can forget about yourself. I battle with these ‘me time’ ideas because I think as a carer or as a mother, your role is to care and to look after, but the self does get overlooked and if you can't get out, if you can't meet other people and you're just one to one with the person you're caring for, it might not be all day but for significant parts of the day, then even though you've got the company of that person, it can be very lonely* (P15: Female, 48, cares for two children with disabilities).

Despite the significance of the need for relatedness to others, the aforementioned extract illustrates that the fulfillment of sociability cannot readily be prioritized over caregiving, perhaps in part due to the moral character of caregiving and the prescriptions of the role (“*I think as a carer or as a mother your role is to care and to look after”*). This finding is in line with research that demonstrates the centrality of morality in perceiving and evaluating ourselves and others (Ellemers et al., 2013; Brambilla and Leach, 2014).

Alongside the limited opportunities for satisfaction of social needs, participants also linked feelings of loneliness and isolation to their restricted ability to look after themselves and pursue leisure activities from which one can derive pleasure.

*When you're looking after someone all the time, you're thinking about them a lot more than you're thinking about your own health, and so that can be really isolating because you stop putting yourself first and you stop looking at what your hobbies are and what makes you happy and things like that* (P04: Female, 24, cares for partner).

### Theme 2: relational losses and deprivations

Loneliness was further linked to the losses and deprivations the caregiver incurred with regard to important close relationships. These deprivations mainly concerned the caregiver's relationship with the cared-for person, especially in cases where this person was a spouse with dementia.

*…the loneliness is there even when I'm with [wife's name] because in reality, I am on my own because she's not relating, there's no conversation other than the weather or the trees, perhaps a bit about the garden, something like that* (P12: Male, 71, cares for wife).

*The worse [partner's name] feels, the more lonely I feel. So he has periods where he doesn't seem like he's communicating with the outside world, so you'll ask him things, he's just very blank, sort of blank, wide eyed stare, not really there* (P04: Female, 24, cares for partner).

But even when the cared-for person's health status did not severely affect the couple's ability to communicate and relate with each other, experiences of loneliness were thought to emerge from the loss of activities and routines that the couple used to enjoy in the past, prior to the onset of illness.

*I'm lucky, I haven't lost [wife's name], I can still speak to [wife's name] and that but we can't do as much as we used to, we can't go out walking the dog together, can't go out riding bikes together. Can't walk around too far. So yes, that's the reason why, because you've got that person. If you're caring for a stranger or somebody who has had their condition for two or three years before you started caring for them, not sounding hard but you don't know that person for the person they used to be. So with a loved one, you lose that person, like I said with [wife's name] and me it's walking, doing bikes and that. With my dad, when mum was ill, in a way he lost his wife because she couldn't talk and recognize him and nothing like that* (P03: Male, 41, cares for wife).

Not only was the relationship, or the shared life, with the cared-for person disrupted severely, but other important relationships within the broader family context were also negatively affected by the caregiving situation. The re-arrangement of these relationships on both practical and emotional levels necessitated by the caregiving situation was sometimes identified as a source of loneliness.

*We rarely go out as a couple, actually that's quite an impact, we rarely go out as a couple these days because it has to be very carefully organized*.

*It can be very lonely and within, my husband and I, I can feel quite lonely there because [name of child with autism] does push a wedge between us and so that's, I feel quite lonely in some of the approaches I make* (P15: Female, 48, cares for two children with disabilities).

A few participants who were caring for an older parent and had themselves become grandparents referred to the deprivations they experienced with regards to the relationship they desired to build and enjoy with their grandchildren. The time that was not presently possible to be invested in these relationships, whilst the grandchildren were still little, due to the caregiving situation was considered invaluable, and to some extent irreplaceable, for the building of memories in the future.

*I'm going to use quite a strong word, I resent not being able to say, “We'll take [grandchild's name] away this weekend”, it's everything has got to be planned and that is a strong word, but I do. [Grandchild's name]'s growing up fast, as children do, he's not going to want to go out with his grandparents for weekends, although having said that his sister did and she's 22 in January and it's lovely, they do want to be with us but we want to do special things with him, which we did with the girls when they were younger and those memories are important, I think. So that's sadly been curtailed and we need to do something about that, I know, but yeah, life has changed* (P08: Female, 69, cares for father).

### Theme 3: social interactions and distancing

Loneliness was not only related to a lack or loss of social relationships, but also to a lack of satisfaction with existing moments of social interaction. Feelings of loneliness were located within social interactions characterized by a lack of understanding, ignorance of the challenges the caregiver faces, and a lack of recognition and acknowledgment of caregivers' contribution, through to a judgmental or even exclusionary stance. Some participants narrated moments of loneliness when they had felt that other people could not genuinely understand them and their situation and did not really know what the caregiver was going through. The loneliness associated with the subjective sense that other people “don't really understand” was described by one participant as a form of “inward loneliness” that persisted despite the building of a network of friends which combatted the “outward loneliness.”

R: …and then I made friends, eventually when we got him into [the name of] School, then that gave me another network but there's the outward loneliness but there's also the inward loneliness as well and I still actually feel quite inwardly lonely.

Int: How does this feel?

R: *It just feels very empty and numb, I feel quite numb sometimes, just how to…I don't want to be self-pitying but it can be very lonely, that people don't really understand* (P15: Female, 48, cares for two children with disabilities).

Social encounters whereby the participants felt that other people unfairly judged them triggered the sense of lack of understanding which, in turn, was linked to feelings of loneliness.

R: *And I feel like people don't understand what's happening with me a lot of the time*.

Int: Why are you saying that?

R: *Because they're not living it and they're not asking about it either. They'll meet [partner's name]. They'll meet up with him and be like oh, he's clearly really ill at the moment, but sometimes I can be quite moody because things are difficult, and I'm tired and I'm working hard. So then people can just think like [partner's name]'s ill and [participant's name]'s just being horrible today, so I'm not going to bother talking to her for a bit and things like that. So yeah it's difficult* (P04: Female, 24, cares for partner).

The inability of others to understand and empathize with caregivers was considered more likely when the illness of the cared-for person was not readily observable, or of a psychological nature, in which case it was thought to be less well understood by the majority of people. Indeed, a mother caring for her son with Attention Deficit Hyperactivity Disorder and high functioning autism narrated her annoyance and wounded feelings when other people questioned the legitimacy of her son's diagnosis and denied the “real existence” of the disorder. This, in turn, challenged her own status as a “caregiver,” an identification that provided her with the legitimacy to seek extra help and support.

Feeling completely understood by others was not nevertheless seen as entirely attainable, unless other people had experienced a similar caregiving situation. For this reason interacting with “similar others” in terms of the caregiving situation and the health status of the cared-for person created a sense of familiarity and comfort among people who could genuinely understand each other.

*That's the other nice thing, when you get talking to people with children that are like yours, you realize it's very different, but very similar if that makes sense but again, it's a comfort to know my child's not the only one who does that, “your child does that as well”, things like the diet and the running off. I remember having a conversation with someone and saying, “[son's name]'s a runner” and she started laughing, she said “I'm sorry, I shouldn't laugh but sometimes you'll say to someone ‘my child is a runner’ and they look at you what you're on about, but I know exactly what you mean”, and that was quite nice because she knows what the runner is!* (P16: Female, 46, cares for son).

Alongside a lack of understanding and empathy, or, even a sense of judgment and subtle condemnation, a lack of recognition and acknowledgment of caregivers' valuable and often “hidden” contribution to the care recipient's well-being was considered to be a further source of loneliness. One participant, who claimed that she did not feel lonely as a person because of the nature of the relationship with her husband, admitted that she was experiencing a form of loneliness that was linked to a lack of recognition of her role as a caregiver. This lack of acknowledgment concerned the world of “non-caregivers” but was also narrated within the context of exchanges with healthcare professionals.

*I know people are busy, I am very aware, incredibly aware how busy these services are in the care services and NHS [National Health Service] but the thought, if only at that front door, ringing the bell, thinking, “that's my primary client, the elderly gentleman/lady needs support but what about the people that are helping them? I need to link with them”, just a couple of words, some acknowledgement, it makes a big difference* (P08: Female, 69, cares for father).

The positioning and “visibility” of the informal caregiver within the healthcare services context were even more problematic when the cared-for person was an adult child suffering from mental illness. Overstretched mental health services, the confidentiality protocols between the patient and healthcare professionals, and the uncertainty around the prognosis of the illness were seen to hamper a fruitful involvement of the caregiver, which would also be sensitive and attentive to the caregiver's informational and emotional needs.

*You are really alone with those feelings because I think as a carer, what you really need to be honest is reassurance that you're doing the right thing and you don't get it. I've never really had it from the recovery service actually, thinking about it now, I never really have had PIP or anybody say, “You're doing a really good job”* (P14: Female, 59, cares for daughter).

Finally, an extreme case of social encounter, characterized by distancing, was offered when a mother narrated an instance of social exclusion linked to her child's health status. This exclusionary social interaction, which triggered a realization that the family was “*very visibly different to the outside world, to the other children”* (P15: Female, 48, cares for two children), was then associated with intense feelings of isolation.

### Theme 4: powerlessness, helplessness, and sole responsibility

Experiences of loneliness were linked to feelings of helplessness and impotence when caregivers faced particular caregiving moments that were experienced as difficult and when help from others was not readily accessible as well as to a general sense of powerlessness to improve the cared-for person's situation. Related to these, a heightened sense of sole responsibility for the cared-for person's welfare was also offered as a context to situate feelings of loneliness.

Although most participants in this study were able to seek and receive satisfying support from family and friends, the inability to “solve” the problems that the person they cared for faced still generated experiences of loneliness. Loneliness, in these instances, was located within a context of powerlessness whereby caregivers lacked control and efficacy.

*I'm really lucky that I've got good friends and family and particularly my stepmother is incredibly supportive. But it's not loneliness in feeling you've got nobody to turn to, it's loneliness in that nobody can really help in a way* (P14: Female, 59, cares for daughter).

*I'm not lonely because of [husband's name] but there are other aspects of being lonely, lonely in terms of feeling isolated and lonely and not being able to find a solution, that sort of loneliness, does that make sense?* (P08: Female, 69, cares for father).

Alongside a general sense of powerlessness, accounts of concrete caregiving moments that were experienced as particularly difficult were also offered to situate experiences of loneliness. The inaccessibility of help from others during these moments and the salience of the caregiver identity as it was being enacted, led to a profound sense of being alone and helpless in the caregiving role, which, in turn, was linked to feelings of loneliness.

*For me, speaking from my own personal thing, I think it's that time when [wife's name] is not well and she's in bed and stays in bed. Sometimes she can have really bad things, stay in a bed for about a day to two days and in that time obviously I won't go out, I'll stay with her. I'll do things but I won't go out. So I think it's that time between me getting up and [wife's name] getting up I think is when I'm loneliest. So yes, I think it's when that time, because your friends aren't there for you then, they're not going to be there for you or they're not going to be there when you've got to get up in the middle of the night and do stuff…so it's those kind of times when you're on your own and it's those times when you can't speak to somebody or they can't come to you* (P03: Male, 41, cares for wife).

*It's almost an instantaneous thing, the loneliness of having to deal with an unexpected problem or a situation, which is usually related to a delusion of some sort and then it passes. Only in the way that perhaps if somebody else was there, you could quite quickly change the focus, you could say “[person's name] is there” or whatever, “[wife's name], why don't you tell so and so about what you did or tell her about the people on the bus?”. But if you're there and trying to deal with that, it's the fact that it's unexpected, you're suddenly thinking on your feet how best to deal with this and you sometimes feel, “I could do with some help”, that's basically really* (P12: Male, 71, cares for wife).

The cared-for person's dependence and reliance on the caregiver and the accompanied sense of sole and exclusive responsibility were occasionally seen to provoke loneliness and an intense realization of ultimately “being on your own.” For instance, a participant, who used to work as a nurse and as a result of this felt confident in looking after her mother, described a form of loneliness she felt when she noticed after her retirement that she could not share anymore the caregiving duties.

*And I think that's what, when I first retired, I did have a sort of loneliness of you can't share the duties. You see I've always been so used to sharing, team work but suddenly you realize that this mum is the person you've got to look after yourself. No-one else is going to put the rubbish out, no-one else is going to change the bed and that can become quite, “Oh dear, why do I have to do everything?”* (P09: Female, 69, cares for mother).

Feelings of loneliness were thus triggered by the lack of presence of others when the others were needed (i.e., moments of helplessness) and an accompanied sense of sole responsibility, but they were also experienced despite the presence of others and provision of their support (i.e., moments of powerlessness), suggesting the multiplicity of circumstances as constitutive of experiences of loneliness.

## Discussion

Designing effective support services and interventions to alleviate loneliness in the caregiving population requires detailed understanding of the phenomenon from the perspective of caregivers themselves. Recognizing a lack of qualitative evidence in this area, the present study sought to build an in-depth, empirically-grounded picture of experiences of loneliness in informal caregivers in a variety of caregiving situations. Consistent with findings from previous qualitative research in the phenomenon of loneliness (Dahlberg, 2007; Stanley et al., 2010; Tiilikainen and Seppänen, 2016), the results of the present study suggest that this psychological state in the caregiving population is similarly complex and multifaceted. Feelings of loneliness were seen to derive from a series of challenges to relationships that threatened caregivers' fundamental need to belong (Baumeister and Leary, 1995). The moral character of the caregiving role that prescribed full attentiveness to the needs of the cared-for person subjected caregivers to the risk of social isolation and diminished social interactions which, at least in part, occasioned feelings of loneliness, as the need for sociability was thwarted (Brambilla and Leach, 2014). It simultaneously shrank caregivers' personal space and time reducing their ability for self-care and leisure. The relational losses and deprivations with regard to significant “Others”—primarily the cared-for person—as well as social encounters that evoked a sense of being ignored, unappreciated, distanced, or even excluded (Williams, 2007) were also thought to generate loneliness, both emotional and social (Weiss, 1973). Lastly, a sense of lack of competence and control over the caregiving situation, of sole responsibility for the cared-for person's welfare, as well as circumstances where the inaccessibility of help was very salient, were linked to experiences of loneliness.

Moreover, the present results allude to the potential contribution of stigma to the generation of experiences of loneliness and isolation in the caregiving population. It was shown that especially in cases whereby the cared-for persons were suffering from psychological conditions, participants experienced covert (e.g., distancing, subtle condemnation) or even overt forms of exclusion. Courtesy stigma describes the stigma that is attached and burdens people who are closely affiliated (e.g., family members, friends) with individuals suffering from stigmatized conditions, such as mental health problems (Goffman, 1963). Courtesy stigma provokes discriminatory behaviors by others with people encountering labeling, stereotyping and separation. The internalization of courtesy stigma by family members, which has been described as affiliate stigma (Mak and Cheung, 2008), leads to negative self-evaluations and behaviors of social withdrawal and concealment of the condition (Ali et al., 2012). Both courtesy and affiliate stigma can therefore deprive caregivers of vital social support, both because social support is not provided or is denied by others and because it is not actively sought by caregivers who withdraw and confine themselves at home (Ntswane and Van Rhyn, 2007; Power, 2008).

Not all circumstances or sources of loneliness are open to change and intervention since many of the grounds of loneliness, such as the relational losses due to incurable illnesses or irreversible health situations, form an unavoidable reality. Yet, the present results suggest that there are aspects of the caregiving experience which could be supported in ways that prevent or alleviate experiences of loneliness. For instance, the heightened risk of social isolation that is conducive to loneliness could be reduced by providing more opportunities for respite care services—or raising awareness of existing possibilities—among the caregiver population that attend not only to the needs of the cared-for person but also to the needs of the caregiver (Ashworth and Baker, 2000). More focused efforts to sensitize the public to the valued contribution of caregivers and the challenges they face could fuel greater social recognition and appreciation of this group and thus also have a role in reducing courtesy and affiliate stigma. Finally, provision for the cared-for-person could be structured in ways that include and value informal caregivers as well as specifically paying attention to caregivers' needs (e.g., informational, emotional). The role of healthcare professionals in this is critical given that the present findings indicate that loneliness in caregiving is sometimes derived from professionals' lack of recognition and support.

### Strengths and limitations of the present study

The limitations of the present study should be considered when interpreting the results. Our sampling strategy sought to recruit a heterogeneous sample of caregivers with respect to their caregiving situation, the illness of the cared-for person and the type of relationship with the cared-for person. The heterogeneity of our sample allowed us to access a wide range of experiences and views and to identify common features in experiences of loneliness linked to a diversity of caregiving situations. Due to this sample heterogeneity however, although the reported themes were clearly identified, we cannot exclude the possibility that additional themes would be detected should further interviews have been conducted with particular sub-groups. The results offered here should therefore be considered as a valid starting point upon which further empirical investigations could be built. Moreover, to our knowledge this is the first study that has attended exclusively to experiences of loneliness linked to a caregiving situation, a phenomenon that is increasingly acknowledged as a considerable challenge of the informal caregiver population (Parsons, 1997; Siriopoulos et al., 1999; Carers UK, 2015a) where the focus of the emerging work is predominantly quantitative (e.g., Beeson, 2003; Ekwall et al., 2005; Soylu et al., 2016). Longitudinal investigations could further be conducted to examine the potential fluctuations and differentiation of the experience of loneliness at different phases of the caregiving journey (e.g., entering the caregiving situation, caring for an individual at terminal stages of illness). A greater focus on the particularities of the experience of loneliness arising from different caregiving situations would be also valuable in the effort to identify risk factors associated with different subgroups of caregivers. Finally, future research would also need to provide accurate estimates of the prevalence of loneliness in this population.

## Conclusion

In an era when healthcare provision shifts away from hospital toward home in order to meet the demands of an increasingly aging population (Christensen et al., 2009) and the growing burden of chronic diseases (Daar et al., 2007), the contribution of informal caregivers is highly significant. Understanding their needs and challenges is crucial for designing suitable support services within the formal healthcare system and in community settings. The present study shed light in one of these challenges, that is, experiences of loneliness, which should be taken into account when interventions that aim to improve the physical and mental health and quality of life of this population are developed and implemented.

## Author contributions

JB, MB, JV, SL, and MW had substantial contribution to the conception of this work. KV and JB designed the study. KV collected and analyzed the data and all authors had substantial contribution to the interpretation of the data. KV drafted a previous version of this article and all authors critically revised it for important intellectual input and finally approved of the version to be published. All authors agree to be accountable for all aspects of the work in ensuring that questions related to the accuracy or integrity of any part of the work are appropriately investigated and resolved.

## Funding

This study is part of a larger research project, titled *Loneliness in the Digital Age (LiDA): Developing Strategies for Empathy and Trust* (grant number: ES/M003558/1). LiDA is financially supported from the Economic and Social Research Council (ESRC) “Empathy and Trust in Communicating Online” (EMoTICON) program, with funding from the Arts and Humanities Research Council (AHRC), the Engineering and Physical Sciences Research Council (EPSRC), the Defence Science and Technology Laboratory (Dstl), and the Centre for the Protection of National Infrastructure (CPNI).

### Conflict of interest statement

The authors declare that the research was conducted in the absence of any commercial or financial relationships that could be construed as a potential conflict of interest.
