# Supplementary material for: Experiences of Loneliness Associated with Being an Informal Caregiver: A Qualitative Investigation
Source: Front Psychol. 2017 Apr 19;8:585. doi: 10.3389/fpsyg.2017.00585 (PMC5395647; doi:10.3389/fpsyg.2017.00585)
Supplement: Supplementary file 1 [file DataSheet1.pdf]

Supplementary Material

Experiences of loneliness associated with being an informal caregiver: a qualitative investigation

Konstantina Vasileiou*, Julie Barnett, Manuela Barreto, John Vines, Mark Atkinson, Shaun Lawson, Michael Wilson

*** Correspondence: Konstantina Vasileiou: k.vasileiou@bath.ac.uk**

1 Supplementary Data

1.1 Interview objectives and protocol

Objectives of the interview

- To explore and understand how carers view and experience their caring role
- To examine whether carers experience loneliness linked to their caring situation and what the nature of this experience is
- To examine management/coping strategies that might be employed to alleviate experiences of loneliness.

1.1.1 Section A: Understanding the caring situation

I wonder if we could start by you telling me a little bit about your experience of caring for your <partner, friend, husband / wife>

Questions

1. Can you please give me a little background as to how you came to take on your caring responsibilities?
   Prompt: How long have you been caring for your <partner, friend, husband / wife>?
   Prompt: Were you able to prepare for taking on this caring role?
2. What do you find particularly challenging about your caring role?
3. Has taking on the role of carer impacted on your own life in any way?
   Prompt: If yes, in what ways has it impacted?

Prompt: Are there any aspects of your life that you have had to limit as a result of your caring role?

4. What kind of support do you receive from family and friends?
5. Do you receive any kind of support from outside agencies?
   Prompt: e.g. NHS, Social Services, respite care.
6. Could you please describe what a typical day is like for you in terms of your caring role?
7. How do you feel about the way your caring role is now a significant part of your life?
   Prompt: What are your thoughts? Feelings?

1.1.2 Section B. Understanding experiences of loneliness and any management strategies employed to ameliorate these experiences.

Questions

1. Have you ever experienced periods of loneliness or felt alone as a consequence of these changes to your lifestyle?
2. Could you tell me about the times when you do feel lonely?
   Prompt: What are your thoughts and feelings during these moments?
   Prompt: How long are these periods of loneliness?
   Prompt: Do you feel that the people around you are aware of your loneliness?
   Prompt: Are there any people you can talk to/confide in?
3. How do you manage to cope during these periods of loneliness?
   Prompt: Please give me some examples of coping strategies.
   Prompt: How are these helpful?
   Prompt: Are there any things that make the loneliness worse?
4. Do you ever use any technologies, such as social media or the internet when you feel lonely?
   Prompt: If yes, what sort of technologies do you use? [If no, why might this be the case?]
   Prompt: Does using these technologies help you feel less lonely?
   Prompt: What do you find helpful with these technologies [or this technology]?
   Prompt: Have you ever felt that using a technology has actually made you feel lonelier or more isolated?
   Prompt: If yes, what happened in that situation?

1.1.3 Section C: Closing the interview

This is all I wanted to ask from my side. Thank you very much for your time! I really appreciate your contribution and I found all the things you said really interesting.

Questions

1. Is there anything else that you would like to add or comment on?
2. Do you have any other observations about how people who provide care for friends and family might feel lonely?

1.2 Screening questionnaire

Demographic Questionnaire

Please fill in this questionnaire if you are interested in taking part in the interview study.

The aim of this questionnaire is to gather some basic information about you and your caring role. This information will help us assess whether people who will express an interest in taking part in the study are eligible for participation. Please, note that all this information will be held and processed in the strictest confidence and in accordance with the Data Protection Act (1998). Only the researcher conducting this study will know your name. Other people involved in this project will not have access to your personal information.

Your background information

Please write down or circle the answer that best describes your view

Questions	Answers
**1. How long have you been providing care?**	Years………………. Months…………………
**2. On a typical day, how long are you responsible for providing care?**	Please estimate the number of hours………………...
**3. Would you say that, as a result of your caring role, you have limited or stopped doing any activities you used to do in the past?**	1. Very much<br>2. Somewhat<br>3. Not really<br>4. Not at all
**4. Are you able to access any respite care?**	1. Yes (If 'yes' how often?……………...…....)<br>2. No
**5. What is your relationship to the person you care for?**	
**6. My gender is:**	1. Female<br>2. Male
**7. My age in years is:**	

**8. What is your highest educational qualification?**	1. Degree or degree equivalent and above<br>2. Higher education to less than degree level (e.g. HND)<br>3. A level Scottish Higher/Vocational level 3 and equivalent<br>4. O level/GCSE/Vocational level 2 and equivalent<br>5. No qualifications
**9. My nationality is:**	
**10. I am:**	1. Single (never married)<br>2. Married (or civil partnership)<br>3. Divorced<br>4. Separated<br>5. Widowed
**11. Do you use the phone to keep in touch with people (family, friends)?**	1. Yes, home landline<br>2. Yes, mobile<br>3. Neither
**12. Do you use digital technologies, such as Facebook or Skype, to keep in touch with people (family, friends)?**	1. Yes<br>2. No
**13. If Yes, what technologies do you use?**	

Thank you very much for your time!

To return this questionnaire please use the free post envelope.

If you have this questionnaire in an electronic format, please send it to the researcher's e-mail address: k.vasileiou@bath.ac.uk
